# Acquisition of Human Polyomaviruses in the First 18 Months of Life

**DOI:** 10.3201/eid2102.141429

**Published:** 2015-02

**Authors:** Rebecca J. Rockett, Seweryn Bialasiewicz, Lebogang Mhango, Jane Gaydon, Rebecca Holding, David M. Whiley, Stephen B. Lambert, Robert S. Ware, Michael D. Nissen, Keith Grimwood, Theo P. Sloots

**Affiliations:** Queensland Children’s Medical Research Institute, Brisbane, Queensland, Australia (R.J. Rockett, S. Bialasiewicz, L. Mhango, J. Gaydon, R. Holding, D.M. Whiley, S. B. Lambert, R.S.Ware, M.D. Nissen, K. Grimwood, T. P. Sloots);; The University of Queensland, Brisbane (R.S. Ware);; Queensland Health, Brisbane (S.B. Lambert);; Pathology Queensland Central Laboratory, Herston, Queensland, Australia (M.D. Nissen);; Griffith University, Gold Coast, Queensland, Australia (K. Grimwood);; Gold Coast University Hospital, Gold Coast (K. Grimwood)

**Keywords:** polyomavirus, viruses, WU polyomavirus, KI polyomavirus, Malawi polyomavirus, Merkel cell polyomavirus, PCR, infectious diseases, community-based study, longitudinal birth cohort

## Abstract

We investigated the presence of 4 human polyomaviruses (PyVs) (WU, KI, Merkel cell, and Malawi) in respiratory specimens from a community-based birth cohort. These viruses typically were acquired when children were ≈1 year of age. We provide evidence that WU, KI, and Malawi, but not Merkel cell PyVs, might have a role in respiratory infections.

Human polyomaviruses (PyVs) JC and BK were discovered in 1971 and are believed to be acquired by a respiratory or fecal–oral route (*1*). They predominantly cause disease in immunocompromised persons (*2*). In the past 7 years, 11 new human PyVs have been described. These include WU (WUPyV), KI (KIPyV), Merkel cell (MCPyV), and Malawi (MWPyV) PyVs, all of which have been detected in respiratory secretions, particularly from children (*3*). Whether these viruses are pathogenic or simply passengers in the respiratory tract is not known. WUPyV and KIPyV were the first respiratory tract–associated PyVs and were discovered in children with acute respiratory infections (*4*,*5*).

MCPyV was identified in Merkel cell carcinoma tissue, and evidence suggested that genome integration of MCPyV initiates cell transformation (*6*). MCPyV has also been reported in respiratory samples, but potential skin or environmental contamination of respiratory samples must be considered (*7*–*9*). In 2013, MWPyV was detected in the fecal sample of a healthy child, and it has also been detected in samples from patients with gastrointestinal symptoms and in anal warts (*10*,*11*). We recently reported that MWPyV was frequently present in respiratory secretions, particularly in children <5 years of age (*12*). However, most of these studies were performed on convenience samples from acutely ill patients and included no samples or limited numbers of samples from healthy controls.

We investigated the presence of the respiratory-associated human PyVs (WUPyV, KIPyV, MCPyV, and MWPyV) in samples collected weekly, regardless of symptoms, from healthy children in Australia during their first 18 months of life. These children were participating in a community-based longitudinal birth cohort study (Observational Research in Childhood Infectious Disease [ORChID]).

## The Study

The ORChID study is an ongoing dynamic birth cohort study in Brisbane (Queensland, Australia) that has been described (*13*). The study was approved by the Human Research Ethics Committees of the Children’s Health Queensland Hospital and Health Service, the Royal Brisbane and Women’s Hospital, and The University of Queensland. In brief, anterior nasal swab specimens were collected at birth and weekly until the child’s second birthday (Technical Appendix).

The present study reports on the first 56 children to complete 18 months (censored for swab specimens collected after 530 study days) of the ORChID study. A total of 3,851 nasal swab specimens (mean 69 swab specimens/child, range 41–77 swab specimens/child) during 29,678 person-days of observation (mean 530 person-days of observation/participant, range 384−560 person-days of observation/participant). These samples were tested for WUPyV, KIPyV, MCPyV, and MWPyV by using reported real-time PCRs (*12*,*13*). Samples were screened for 13 common respiratory viruses according to the ORChID study protocol (Technical Appendix).

A sole detection episode was defined as ≥1 consecutive swab specimens in which an individual PyV was the only virus detected, and no other viruses were reported 7 days before or after detection of the PyV. Detection of a different PyV or the same PyV after 30 days and ≥2 intervening negative samples was considered a new infection episode. During the period of the sole detection episode, clinical symptoms were broadly categorized as upper respiratory, lower respiratory, nonspecific, and gastrointestinal (Technical Appendix). Nonspecific symptoms were only separately categorized when unaccompanied by upper or lower respiratory symptoms. Gastrointestinal symptoms were recorded in the presence or absence of respiratory symptoms.

All 4 novel PyV viruses were detected in respiratory samples from children ≤18 months of age; MWPyV was the predominant virus (157 positive detections) (Table 1). A total of 23% (13/56 for MCPyV) and 56% (31/56 for MWPyV) of children had ≥1 positive result for 1 of the PyVs. WUPyV, KIPyV, and MWPyV were initially detected when the child was ≈1 year of age, and each virus was detected for a mean of 2 consecutive weeks after primary detection. MCPyV was detected less frequently; primary detections occurred in children ≈7 months of age, which was earlier than detection of WUPyV, KIPyV, and MWPyV (p<0.001, by Mann-Whitney 2-tailed test with pairwise comparisons of ages). MCPyV was not seen in any consecutive swab specimen collections.

**Table 1:** Detection of polyomavirus by real-time PCR in a subset of 56 ORChID study participants who had weekly anterior nasal swab specimens collected for 18 months during 2010–2014*

Characteristic	**WUPyV**	**KIPyV**	**MCPyV**	**MWPyV**
No. detections (3,851 nasal swab specimens)	36	63	13	157
No. positive/no. tested (%)	14/56 (25)	26/56 (45)	13/56 (23)	31/56 (56)
Median age at primary detection, mo (range)	11 (5.2–16.6)	10 (5.6–18.6)	7 (0.07–18.8)	13 (0.9–13.1)
Median length of viral shedding, wk (range)	2 (1–4)	2 (1–5)	1 (1)	1.2 (1–3)
Co-detection with 13 other respiratory viruses (%)	18/36 (50)	31/63 (49)	3/13 (21)	52/157 (33)
Median cycle threshold (range)†	29.45 (18–37)	30.91 (18–40)	38.58 (34–40)	35.15 (30–40)

*ORChID, Observational Research in Childhood Infectious Diseases; WUPyV, WU polyomavirus; KIPyV, KI polyomavirus; MCPyV, Merkel cell polyomavirus; MWPyV, Malawi polyomavirus. **†Cycle thresholds are approximately inversely related to viral load.**

WUPyV (50%, 18/36) and KIPyV (49%, 31/63) were commonly detected with other respiratory viruses. MWPyV (33%, 52/157) and MCPyV (21%, 3/13) had lower semiquantitative viral loads (p<0.001, by Mann-Whitney 2-tailed test with pairwise comparisons of cycle thresholds), and were less frequently detected with other respiratory viruses (Table 1; Technical Appendix).

Symptoms were reported during sole detection episodes for WUPyV, KIPyV, and MWPyV, but not for MCPyV (Table 2). During symptomatic episodes, numerous overlapping respiratory virus detections were commonly observed, which made sole detection episodes of PyV too rare to be considered in a formal statistical analysis. However, sole detection episodes corresponded to parental reporting of clinical symptoms for 57% (4/7) of WUPyV and 36% of KIPyV (5/14) and MWPyV (13/36) infection episodes (Table 2). Most symptoms reported during the sole detection episodes were upper respiratory (Table 2).

**Table 2:** Characteristics of persons infected with polyomavirus in a subset of 56 ORChID study participants who had weekly anterior nasal swab specimens collected for 18 months during 2010–2014*

Characteristic	**WUPyV**	**KIPyV**	**MWPyV**
No. sole detection episodes	9	14	36
No. sole detection episodes with symptoms/no. tested, %	4/7 (57)	5/14 (36)	13/36 (36)
No. sole detection episodes without symptoms	3	9	23
Symptoms of LRTI	0	0	0
Symptoms of URTI	4	3	9
Symptoms of URTI and LRTI	0	0	2
Only nonspecific symptoms	0	1	2
Diarrhea/vomiting	1	1	1
Symptoms not available	2	0	0

*ORChID, Observational Research in Childhood Infectious Diseases; WUPyV, WU polyoma virus; KIPyV, KI polyomavirus; MWPyV, Malawi polyomavirus; LRTI, lower respiratory tract infection; URTI, upper respiratory tract infection.

## Conclusions

Primary acquisition of WUPyV, KIPyV, and MWPyV occurred most commonly when children were ≈12 months of age, which is consistent with previous serologic data showing PyV primary infection in children at an early age (*3*). MCPyV was also detected within this cohort, but age, frequency of detection, and lower levels of viral shedding contrasted with the findings for WUPyV, KIPyV, and MWPyV. This finding is supported by other retrospective studies that showed that MCPyV is more frequently detected in the respiratory tract of adults (*7*). Previously reported high co-detection rates of WUPyV and KIPyV with other respiratory viruses (>70%) have hampered efforts to associate these viruses with clinical symptoms (*3*,*14*). However, we observed a lower rate of PyV co-detection, which enables us to examine the association of PyV sole detection episodes with symptoms. This examination showed that most symptoms were upper respiratory, although gastrointestinal symptoms were also reported during WUPyV, KIPyV, and MWPyV sole detection episodes.

A higher viral load was observed for WUPyV and KIPyV than for MWPyV and MCPyV. Although use of cycle thresholds as a semiquantitative marker has some limitations, thresholds suggest that WUPyV and KIPyV actively replicate in the respiratory tract and may account for the higher rate of reported symptoms with sole detection of WUPyV. A previous study in the Netherlands also reported a higher symptom association of WUPyV than KIPyV, but MWPyV was not investigated in that study (*15*). MWPyV was first detected in fecal specimens from healthy children, but we found that MWPyV was the most prevalent PyV detected in respiratory specimens and that it was associated with upper respiratory infection symptoms in >33% of sole detection episodes. We found that 1/36 participants had gastrointestinal symptoms during an episode of MWPyV sole detection, which is an observation that warrants further investigation.

MCPyV is shed from healthy skin, and MWPyV has been detected in anal warts. Thus, a limitation of this study is potential cutaneous contamination of swab specimens by the parent or child during sample collection. Although the complex nature of virus acquisition and overlapping intervals of virus detection confound the association of PyV sole detection episodes with particular symptoms, our data show that WUPyV, KIPyV, and MWPyV, but not MCPyV, are frequently detected within the respiratory tract of healthy children <18 months of age and are associated with mild upper respiratory symptoms.

**Technical Appendix.** Supplementary methods for detection of human polyomaviruses in the first 18 months of life.
