# Supplementary material for: Acquisition of Human Polyomaviruses in the First 18 Months of Life
Source: Emerg Infect Dis. 2015 Feb;21(2):365–7. doi: 10.3201/eid2102.141429 (PMC4313659; doi:10.3201/eid2102.141429)
Supplement: Supplementary file 1 — Technical Appendix. Supplementary methods for detection of human polyomaviruses in the first 18 months of life. [file 14-1429-Techapp-s1.pdf]

Article DOI: http://dx.doi.org/10.3201/eid2101.141429

Acquisition of Human Polyomaviruses in the First 18 Months of Life

Technical Appendix

Supplementary Methods for Detection of Human Polyomaviruses in the First 18 Months of Life

Observational Research in Childhood Infectious Diseases Study Design

In brief, 165 healthy full-term babies were enrolled from birth after initially approaching their parents while they were attending antenatal clinics in Brisbane, Queensland, Australia. Parents were taught to collect anterior nasal swab specimens from their infant beginning from birth and continuing weekly until the child's second birthday. The swab specimens were then mailed directly to the research laboratory. In addition, a daily symptom diary was recorded and returned monthly. Throughout the study, research staff maintained regular contact with the families.

Quality Control of Nucleic Acid Extraction

To monitor the efficiency and reproducibility of DNA extraction, samples were spiked with 1×10^4 copies of equine herpes virus DNA (PCR crossing point = 30 cycles) before DNA extraction (*1*). Samples with a crossing point ≥3 of 30 cycles were considered efficiently extracted and free of PCR inhibitors. Samples that did not satisfy this requirement were re-extracted.

Respiratory Virus PCR

Respiratory virus screening used nucleic acid pools to test for respiratory syncytical virus; influenza A and B viruses; parainfluenza 1, 2 and 3 viruses; human adenovirus; human metapneumovirus; coronaviruses 229E, HKU1, OC43, and NL63; human bocavirus; and WU polyomavirus (WUPyV), KI polyomavirus (KIPyV), Merkel cell polyomavirus (MCPyV), and Malawi polyomavirus (MWPyV) by using previously reported real-time PCRs (*2*,*3*).

Primers and probes specific for MWPyV have 100% homology to viral protein 1 sequences reported for the highly homologous MWPyV species variants HPyV10 and MXPyV. Analytical sensitivities for the 2 polyomavirus assays used in this study (assay 1: MWPyV and MCPyV; assay 2: WUPyV and KIPyV) were <10 copies/µL of extract. Rhinovirus testing was performed on individual extracts because of the anticipated high number of positive detection results. Cycle thresholds were used as semiquantitative markers of viral load as described (*4*).

Respiratory Symptom Categories

During the period of detection, clinical data were broadly categorized as upper respiratory (ear infection, runny nose, cough, sore throat and muscle aches), lower respiratory (wheezing, shortness of breath, pulmonary congestion and pneumonia), nonspecific (fever, chills, headaches, irritability, and decreased activity), and gastrointestinal (>3 lose stools in a 24-hour period and any vomiting).

References

<jrn>1. Bialasiewicz S, Whiley DM, Buhrer-Skinner M, Bautista C, Barker K, Aitken S, et al. A novel gel-based method for self-collection and ambient temperature postal transport of urine for PCR detection of *Chlamydia trachomatis*. Sex Transm Infect. 2009;85:102–5. PubMed http://dx.doi.org/10.1136/sti.2008.032607</jrn>

<jrn>2. Rockett RJ, Sloots TP, Bowes S, O'Neill N, Ye S, Robson J, et al. Detection of novel polyomaviruses, TSPyV, HPyV6, HPyV7, HPyV9 and MWPyV in feces, urine, blood, respiratory swabs and cerebrospinal fluid. PLoS ONE. 2013;8:e62764. PubMed http://dx.doi.org/10.1371/journal.pone.0062764</jrn>

<jrn>3. Lambert SB, Ware RS, Cook AL, Maguire FA, Whiley DM, Bialasiewicz S, et al. Observational Research in Childhood Infectious Disease s (ORChID): a dynamic birth cohort study. BMJ Open. 2012;2:pii: e002134.</jrn>

<jrn>4. Christensen A, Nordbø SA, Krokstad S, Rognlien AG, Døllner H. Human bocavirus commonly involved in multiple viral airway infections. J Clin Virol. 2008;41:34–7. PubMed http://dx.doi.org/10.1016/j.jcv.2007.10.025</jrn>
